# A mutualistic endophyte alters the niche dimensions of its host plant

**DOI:** 10.1093/aobpla/plv005

**Published:** 2015-03-10

**Authors:** Melanie R. Kazenel, Catherine L. Debban, Luciana Ranelli, Will Q. Hendricks, Y. Anny Chung, Thomas H. Pendergast, Nikki D. Charlton, Carolyn A. Young, Jennifer A. Rudgers

**Affiliations:** 1 Department of Biology, University of New Mexico, Albuquerque, NM 87131, USA; 2 Rocky Mountain Biological Laboratory, Crested Butte, CO 81224, USA; 3 Department of Biology, University of Virginia, Charlottesville, VA 22904, USA; 4 Division of Science and Mathematics, University of Minnesota, Morris, Morris, MN 56267, USA; 5 Forage Improvement Division, The Samuel Roberts Noble Foundation, Ardmore, OK 73401, USA; 6 Department of Crop and Soil Sciences, University of Georgia, Athens, GA 30602, USA

**Keywords:** *Epichloë*, fungal endophyte, mutualism, *Poa leptocoma*, *Poa reflexa*, symbiosis

## Abstract

Mutualisms can play important roles in influencing species coexistence and determining community composition. However, few studies have tested whether such interactions can affect species distributions by altering the niches of partner species. In subalpine meadows of the Rocky Mountains, USA, we explored whether the presence of a fungal endophyte (genus *Epichloë*) may shift the niche of its partner plant, marsh bluegrass (*Poa leptocoma*) relative to a closely related but endophyte-free grass species, nodding bluegrass (*Poa reflexa*). Using observations and a 3-year field experiment, we tested two questions: (i) Do *P. leptocoma* and *P. reflexa* occupy different ecological niches? and (ii) Does endophyte presence affect the relative fitness of *P. leptocoma* versus *P. reflexa* in the putative niches of these grass species? The two species were less likely to co-occur than expected by chance. Specifically, *P. leptocoma* grew closer to water sources and in wetter soils than *P. reflexa*, and also had higher root colonization by mycorrhizal fungi. Endophyte-symbiotic *P. leptocoma* seeds germinated with greater frequency in *P. leptocoma* niches relative to *P. reflexa* niches, whereas neither endophyte-free (experimentally removed) *P. leptocoma* seeds nor *P. reflexa* seeds showed differential germination between the two niche types. Thus, endophyte presence constrained the germination and early survival of host plants to microsites occupied by *P. leptocoma*. However, endophyte-symbiotic *P. leptocoma* ultimately showed greater growth than endophyte-free plants across all microsites, indicating a net benefit of the symbiosis at this life history stage. Differential effects of endophyte symbiosis on different host life history stages may thus contribute to niche partitioning between the two congeneric plant species. Our study therefore identifies a symbiotic relationship as a potential mechanism facilitating the coexistence of two species, suggesting that symbiont effects on host niche may have community-level consequences.

## Abstract (precis)

Few studies have tested whether mutualisms may affect species distributions by altering the niches of partner species. We show that a fungal endophyte is associated with a shift in the soil moisture niche of its host plant relative to a co-occurring, endophyte-free congener. The endophyte appeared to initially restrict its host's distribution to wetter microsites before positively affecting its growth, suggesting the value of considering symbiont effects at different partner life stages. Our study identifies a symbiotic relationship as a potential mechanism facilitating the coexistence of two species, suggesting that symbiont effects on host niche may have community-level consequences.

## Introduction

Mutualisms can play important roles in influencing species coexistence and determining community composition (Palmer *et al.* 2003; Umbanhowar and McCann 2005; Jones *et al.* 2012). Such positive interactions can alter species' range sizes by expanding or shifting the niches of individual partners (Bruno *et al.* 2003; Poisot *et al.* 2011). In addition, mutualists may increase the competitive dominance of host species, thereby decreasing species coexistence and the species richness of competitors in the community (Hartnett and Wilson 1999; Rudgers *et al.* 2007). In other systems, mutualisms appear to increase species diversity if they benefit multiple community members or preferentially benefit rare species (Müller and Godfray 1999; Hart *et al.* 2003).

A small number of studies have tested whether mutualisms have the potential to influence species coexistence by altering the niches of partner organisms. Considerable niche overlap among species is predicted to result in competitive exclusion; mutualisms may bring about niche shifts or changes in competitive hierarchies that allow for species coexistence (Bruno *et al.* 2003). For instance, mutualistic ants were found to shift the niche of *Lycaeides melissa* caterpillars, increasing their survival on a novel, sub-optimal host plant by providing protection from predators (Forister *et al.* 2011). In some plants, arbuscular mycorrhizal (AM) fungi may increase species coexistence by reducing the ability of one plant species to repress the other via competition for shared soil resources (Wagg *et al.* 2011). An endosymbiont was found to affect the long-distance airborne dispersal of the *Erigone atra* spider, decreasing the spider's dispersal range and thereby limiting the spider's niche (Goodacre *et al.* 2009). In contrast, ant-mediated dispersal of *Hexastylis arifolia* seeds was not found to result in plant niche expansion via enhanced resource utilization or decreased density dependence (Warren *et al.* 2010). Although not all mutualisms bring about niche alteration, these positive interactions may affect the niche dimensions of partners in some systems.

This study focused on the potential role of endophytic fungi in influencing host plant niche dimensions. Endophytic fungi and bacteria are ubiquitous symbionts of plant tissues (Bacon and White 2000) and can range from mutualists to pathogens. Certain endophytes are responsible for the production of secondary metabolite compounds previously attributed to plants (Strobel 2002) and can buffer their hosts against damage by pathogens and herbivores (Clay and Schardl 2002) as well as abiotic stress (Rodriguez *et al.* 2008). Systemic, vertically transmitted fungal endophytes, which commonly form symbioses with cool-season grasses, often act as mutualists and are known to improve host tolerance to drought, increase plant nitrogen and phosphorus content, and produce alkaloids that deter herbivores (Malinowski and Belesky 2000; Clay and Schardl 2002; Cheplick and Faeth 2009; Schardl 2010). Such effects on host plants can in turn have community- and ecosystem-level consequences, influencing processes such as plant succession (Rudgers *et al.* 2007) and decomposition (Lemons *et al.* 2005; Omacini *et al.* 2012). However, whether an endophyte can shift the niche of its host and thereby alter host interactions with closely related competitor plants remains undocumented.

We evaluated whether the presence of a fungal endophyte may alter the niche of *Poa leptocoma* (marsh bluegrass) and affect its co-occurrence with a similar but naturally endophyte-free grass species, *Poa reflexa* (nodding bluegrass) in subalpine meadows of the Rocky Mountains, USA. Although it has been suggested that *P. leptocoma* prefers wetter habitats (Barkworth *et al.* 2007), no data exist that document niche characteristics of the two species. For our study, we conceptualized the ‘niche’ of a species as the distinctive set of abiotic and biotic microsite conditions associated with a species' current distribution (Levine and HilleRisLambers 2009). We surveyed *P. leptocoma* and *P. reflexa* populations to document endophyte symbiosis and carried out endophyte species characterization via DNA sequencing. We then addressed two questions to explore the effect of endophyte symbiosis on multiple stages of host life history: (i) Do *P. leptocoma* and *P. reflexa* occupy different ecological niches? and (ii) Does endophyte presence affect the relative fitness of *P. leptocoma* versus *P. reflexa* in their putative niches? We hypothesized that (i) the two species occupy different ecological niches in the habitats examined, and that (ii) endophyte symbiosis alters the relative fitness of *P. leptocoma* versus *P. reflexa* in the two species' putative niches. We predicted that endophyte-symbiotic *P. leptocoma* seeds would perform best in *P. leptocoma* microsites (niches), and that *P. reflexa* seeds would perform best in *P. reflexa* microsites. If the endophyte were to shift the niche dimensions of its host, then endophyte-free *P. leptocoma* individuals would have niche requirements more similar to those of *P. reflexa*; thus, we predicted that *P. leptocoma* endophyte-free seeds would perform better in *P. reflexa* microsites than in *P. leptocoma* microsites.

## Methods

### Study organisms

Both *P. leptocoma* (sect. Oreinos) and *P. reflexa* (sect. Homalopoa) grow in western North America in subalpine meadow and forest understory environments near streams (Barkworth *et al.* 2007). *Poa leptocoma* has scabrous panicle branches, whereas *P. reflexa* has smooth branches (Shaw 2008), and it is difficult to differentiate between the two species prior to flowering. Thus, naturally occurring plants used in the study were identified while they were flowering.

We examined the symbiosis between *P. leptocoma* and a systemic, vertically transmitted fungal endophyte in the genus *Epichloë. Epichloë* spp. are plant-symbiotic fungi in the family Clavicipitaceae and occur in ∼20–30 % of all grass species (Leuchtmann 1992; Rodriguez *et al.* 2009). Fungal endophytes can be transmitted to a host plant horizontally via spores and/or vertically from plant to seed (Clay and Schardl 2002); the genus *Epichloë* includes non-hybrid sexual species (horizontal and some vertical transmission) and hybrid and non-hybrid asexual species, formerly called *Neotyphodium* spp. (vertical transmission) (Leuchtmann *et al.* 2014).

### Endophyte frequency and species characterization

To evaluate endophyte symbiosis in the two *Poa* species, we collected leaf samples from *P. leptocoma* and *P. reflexa* populations in the Rocky Mountains of Colorado, USA, from the Rocky Mountain National Park and surrounding areas (Larimer, Grand and Boulder counties) south to Gunnison County [**see** **Supporting Information**]. Thin sections from the inner leaf sheaths of collected samples were stained with aniline blue lactic acid dye following the methods in Bacon and White (1994). Stained sections were checked for endophyte presence using a microscope at ×200 magnification.

We determined *Epichloë* species diversity present in *P. leptocoma* using plant material collected from six sites in Gunnison County, CO [**see** **Supporting Information**]. Total DNA was extracted from *P. leptocoma* plant material using a QIAGEN MagAttract 96 DNA Plant Core Kit (QIAGEN Inc., Valencia, CA, USA). Multiplex PCR was carried out using 19 established genus-specific primers representing housekeeping, mating type and key alkaloid biosynthesis genes (Charlton *et al.* 2012; Takach *et al.* 2012) to determine endophyte presence and alkaloid production potential.

We identified species by direct sequencing the housekeeping genes *tefA* and *tubB*, using primer sets tef1-exon1d-1 and tef1-exon6u-1 for *tefA* and *tubB* F1 (5′ CTCTGTTTGTCTTGGGGACC 3′) and T1.2 for *tubB* (Craven *et al.* 2001; Young *et al.* 2005; Charlton *et al.* 2012). Sequences representing *Epichloë* species diversity were analysed using phylogeny.fr with default settings (Dereeper *et al.* 2008, 2010). Sequences were aligned using MUSCLE (version 3.7) (Edgar 2004), phylogenetic trees were inferred by maximum likelihood using PHYML (version 3.0) (Guindon and Gascuel 2003) with an approximate likelihood ratio test (Anisimova and Gascuel 2006), and the trees were rendered with TREEDYN (version 198.3) (Chevenet *et al.* 2006). We annotated clades in accordance with Leuchtmann *et al.* (2014).

### Do *P. leptocoma* and *P. reflexa* occupy different ecological niches?

#### Surveys

To document the current distributions of the bluegrass species, we marked naturally occurring plants at four sites. First, during August 2008, we marked 77 *P. leptocoma* plants and 40 *P. reflexa* plants near a stream system in Virginia Basin, Gunnison County, CO (38°58.314N, 106°58.942W, elevation 3229 m). The nearest individual of each species was marked approximately every 2 m along transects placed in three regions of the watershed: directly in and along a shady streambed (where only *P. leptocoma* was present), next to a sunny streambed (both species) and in a wet meadow between the two streams (both species). Then, during August 2011, we used the same transect method to mark a minimum of 10 individuals per species at each of two sites near Schofield Pass (Schofield and Maroon, 39°01.329N, 107°02.938W, elevation 3218 m) and at one site on Snodgrass Mountain (38°55.083N, 106°59.175W, elevation 3357 m) [**see** **Supporting Information** **for sample sizes**].

#### Co-occurrence

To examine natural patterns of co-occurrence, we compared *P. leptocoma* and *P. reflexa* densities in randomly placed quadrats versus quadrats placed around target plants of each species (24–25 July 2012). For the random samples, we placed 30 × 30 cm quadrats at 2 m intervals along eight transects in the Virginia Basin study area and in nearby Copper Creek (38°58.01N, 106°58.17W). We counted the number of flowering *P. leptocoma* and *P. reflexa* individuals per quadrat to determine an expected density per square metre for reproductive adults of each species, evaluating a total of 58 quadrats. At each transect interval, we also located the nearest bluegrass individual (*P. leptocoma* or *P. reflexa*), placed the quadrat around the centre of the identified target plant and counted the number of flowering individuals of each species in the quadrat to quantify plant density. We evaluated a total of 24 *P. leptocoma* targeted quadrats and 25 *P. reflexa* targeted quadrats. We used a one-tailed, unequal variance *t*-test to compare the density of each species in the randomly located quadrats against their densities in the targeted quadrats. These analyses tested (i) whether targeted quadrats had higher densities of conspecifics than expected by chance, indicative of a clumped species distribution and (ii) whether the two species were less likely to co-occur than expected by chance, suggestive of different niches or competitive exclusion.

#### Niche dimensions

As anecdotal data suggested that *P. leptocoma* prefers wetter environments relative to *P. reflexa* (Barkworth *et al.* 2007), we examined water availability at four survey sites to describe the abiotic microhabitats of the two bluegrasses. During 8–10 August 2011, we measured the distance of each marked plant (to the nearest 0.01 m) to the nearest source of water (a stream or snow bank, measuring to the closer of the two). We also measured soil volumetric water content (VWC) at each plant using a soil moisture metre (HydroSense meter, Campbell Scientific, Inc., Logan, UT, USA) with 12 cm probes, consistent with rooting depth. At the Virginia Basin site, we additionally measured soil moisture at marked plants approximately weekly during the 2011 growing season following snowmelt on 12 July 2011, excluding eight plants in locations too rocky to obtain accurate soil moisture data [**see** **Supporting Information** **for sample sizes**].

To evaluate one biotic niche dimension, we examined *P. leptocoma* and *P. reflexa* roots for colonization by fungal symbionts other than *Epichloë* spp., which do not occupy roots. Root fungi were of particular interest because *Epichloë* presence has been shown to reduce mycorrhizal colonization in grass hosts (Mack and Rudgers 2008). During 8–12 August 2012, we collected roots from marked plants at the four survey sites. Roots were stored in 70 % ethanol, cleared with 10 % KOH and stained with trypan blue (Phillips and Hayman 1970). We mounted stained roots onto glass slides and assessed fungal colonization using the magnified intersection method of McGonigle *et al.* (1990). At ×400 magnification, we noted the presence of mycorrhizal hyphae, vesicles and arbuscules, as well as dark septate endophyte hyphae and microsclerotia, at each root intersection.

#### Statistical analysis: niche characteristics

We analysed niche characteristics with ANOVA, including the factor of plant species identity, the random effect of site and the site × species interaction (SAS version 9.2, SAS Institute, Inc., Cary, NC, USA). A significant species × site interaction would indicate inconsistency in species niche characteristics across sites. When such interactions were significant, we used *a priori* orthogonal contrasts to compare species within each site. We log-transformed the responses of distance to water and VWC to improve normality of residuals and homogeneity of variances.

### Does endophyte presence affect the relative fitness of *P. leptocoma* versus *P. reflexa* in their putative niches?

#### Experimental approach

We used the marked plants at the Virginia Basin site as replicates of each species' specific microhabitat, i.e. its putative niche. This approach eliminated the need to replicate the precise abiotic and biotic conditions characteristic of each species' microhabitat. To test whether plant performance differed between species and/or with endophyte symbiosis, we planted seeds into natural *P. leptocoma* and *P. reflexa* microhabitats and compared germination, seedling survival and plant growth.

#### Seed types and sources

Surrounding each marked, naturally occurring plant, we planted a total of nine seeds: three naturally endophyte-free (E−) *P. reflexa* seeds collected from field plants; two naturally endophyte-symbiotic (E+) *P. leptocoma* seeds collected from field plants (‘E+ natural’); three endophyte-free (E−) *P. leptocoma* seeds collected from greenhouse-grown plants from which the endophyte was experimentally removed; and one *P. leptocoma* endophyte-symbiotic (E+) ‘control’ seed collected from a greenhouse-grown plant. This approach controlled for maternal effects by decoupling endophyte presence from effects associated with plant genotype or maternal environment.

For the *P. leptocoma* E+ natural and *P. reflexa* seed treatments, we collected seeds from the Virginia Basin site during late August 2009 and stored them at 4 °C until planting. *Poa leptocoma* E− seeds and E+ control seeds came from plants grown in a common greenhouse environment from the Virginia Basin seed stock. To create endophyte-free (E−) *P. leptocoma* seeds, we experimentally removed the endophyte by germinating seeds for 5 weeks on wet filter paper in standard plastic Petri plates containing the fungicide benomyl (2 g/L). Seeds for the *P. leptocoma* E+ control treatment were germinated in Petri plates that contained water only. Following germination, *P. leptocoma* E+ control and E− seedlings were transplanted into 2.5 × 4 cm plastic pots and grown for 2–6 weeks on a light bench. Once seedlings had established a root system, they were transplanted into 10 cm^2^ pots with a 1 : 1 mixture of Pro-mix BX (Premier Horticulture, Quakertown, PA, USA) and sterile sand and grown until reproductive. Endophyte status of each plant was confirmed microscopically following the methods in Bacon and White (1994). Seeds for the experiment were collected in the greenhouse during July 2010.

#### Experimental methods

Prior to planting, we lightly glued seeds to plastic toothpicks (Wright *et al.* 2006) with water-soluble glue (Elmer's Washable School Glue, Elmer's Products, Inc., Columbus, OH, USA). We used a different toothpick colour for each type of seed, and planted the toothpicks in the ground to position the seeds just at the soil surface. The seeds (*N* = 920) were planted on 9 July 2011 in randomized order, forming a circle with a radius of ∼5 cm around each original, naturally occurring plant [**see** **Supporting Information**]. This spacing mimicked realistic field seedling densities.

In 2011, we scored seed germination weekly for 7 weeks. After allowing the plants to overwinter, we scored germination, measured the height of the tallest leaf (cm) and counted the number of leaves and tillers on each plant twice in 2012 (14 June and 20 August) and twice in 2013 (20 June and 2 September). On each of these dates, for seeds that had previously germinated, we also recorded plant survival. On 2 September 2013, we harvested all remaining plants and counted the total number of leaves and the number of live leaves on each plant. We rinsed the roots of harvested plants to remove soil, divided each plant at the soil interface into aboveground and belowground biomass and dried the samples at 60 °C. Following drying, we weighed samples using a microbalance (Sartorius Cubis MSE 3.6P, Data Weighing Systems, Inc., Elk Grove, IL, USA).

##### Statistical analysis

Analyses were designed to test our predictions that *P. leptocoma* E+ seeds would perform best in *P. leptocoma* microsites, *P. reflexa* seeds would perform best in *P. reflexa* microsites and endophyte removal from *P. leptocoma* would shift the species' niche toward *P. reflexa* microsites. All models included the fixed effects of seed identity, microsite (*P. leptocoma* or *P. reflexa* niche) and their interaction, as well as the random effect of plant identity (nested within microsite) to account for the non-independence of seeds that were placed around the same naturally occurring plant. To test *a priori* hypotheses, we constructed contrasts to compare performance between the *P. leptocoma* and *P. reflexa* niches within each seed type. We also specifically compared the responses of greenhouse-grown seeds (*P. leptocoma* E+ control versus *P. leptocoma* E−) within each microsite.

We analysed germination and survival data with a log-linear model, including the binomial response (0/1) with a logit link function (Proc GLIMMIX, SAS version 9.2, SAS Institute, Inc.). For the subset of seeds that germinated, we analysed the time to germinate (in weeks), and for the subset of seedlings that died, we analysed survival time (in weeks) using generalized linear models with a Gaussian distribution. Growth responses (number of live leaves at the time of harvest, aboveground biomass and belowground biomass) were analysed using MANOVA followed by univariate analysis. Aboveground and belowground biomass data were log-transformed, and live leaf data were square-root-transformed. All models included the fixed effects of seed identity, microsite (*P. leptocoma* or *P. reflexa* niche) and their interaction, as well as the random effect of plant identity (nested within microsite) to account for the non-independence of seeds that were placed around the same naturally occurring plant. To test *a priori* hypotheses, we constructed contrasts to compare performance between the *P. leptocoma* and *P. reflexa* niches within each seed type. We also specifically compared the responses of greenhouse-grown seeds (*P. leptocoma* E+ control versus *P. leptocoma* E−) within each microsite. For growth responses, *P. leptocoma* E+ control and *P. leptocoma* E+ natural data were combined because few individuals were alive at the time of harvest.

## Results

### Endophyte frequency and species characterization

In our field surveys, *Epichloë* species approached 100 % frequency in *P. leptocoma* [**see** **Supporting Information**]. No systemic endophyte was found in *P. reflexa.* In greenhouse-grown seedlings, the endophyte was vertically transmitted to 95 % of established seedlings (J. A. Rudgers, unpubl. data).

Examined samples contained single copies of endophyte *tubB* and *tefA* genes, indicating the presence of a non-hybrid *Epichloë* species in *P. leptocoma*. Phylogenies revealed that all *tubB* and *tefA* sequence data grouped in the *Epichloë typhina* subspecies *poae* subclade [**see** **Supporting Information**]. Sexual *Epichloë* spp. are heterothallic, requiring individuals of two mating types for successful reproduction; all examined samples were positive for *mtBA* markers, suggesting that the examined populations of *P. leptocoma* endophytes comprise a single mating type, *MTB*. While it is possible that the examined *P. leptocoma* endophytes are capable of sexual reproduction, we saw no evidence of both mating types within the populations we evaluated. In addition, stromata were not observed in field or greenhouse-grown plants, further suggesting that the examined endophytes likely represent asexual, non-hybrid populations of *E. typhina* subsp. *poae*.

We also found genes linked to alkaloid production within the *E. typhina* subsp. *poae* populations. All samples were positive for *perA* (*PER* locus required for peramine production) markers, indicating the potential to produce peramine, a known insect feeding deterrent. All samples from the Washington Gulch population and half of samples from the Slate River population also contained ergot alkaloid biosynthesis genes (*EAS* locus), indicating the potential to produce the ergot alkaloid intermediate chanoclavine. All samples examined from the Virginia Basin site contained identical endophyte genotypes and were positive for *PER* markers but negative for *EAS* markers.

### Do *P. leptocoma* and *P. reflexa* occupy different ecological niches?

#### Co-occurrence

**Figure 1.**
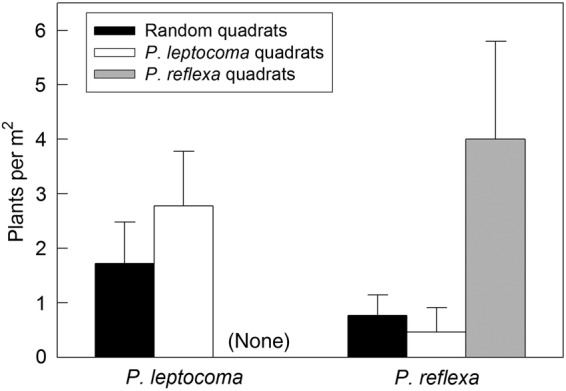
Natural densities of *P. leptocoma* and *P. reflexa* individuals in Gunnison Co., CO, USA, sampled in three quadrat types: randomly placed quadrats along transects, quadrats centred on a focal *P. leptocoma* individual, and quadrats centred on a focal *P. reflexa* individual. Bars show the mean number of individuals per square metre (excluding the focal plant) with s.e.

Our species distribution surveys indicated low local co-occurrence of the two *Poa* species. *Poa leptocoma* individuals were absent from quadrats placed around target *P. reflexa* individuals, and in the randomly placed quadrats were significantly less likely to co-occur with *P. reflexa* than with a conspecific (Fig. 1, *t*(23) = −2.77, *P* = 0.005) or than expected by chance (Fig. 1, *t*(23) = −2.34, *P* = 0.014). *Poa reflexa* was significantly clumped relative to the randomly sampled quadrats (Fig. 1, *t*(24) = −1.76, *P* = 0.045), and significantly more likely to co-occur with a conspecific target than with *P. leptocoma* (Fig. 1, *t*(24) = −1.92, *P* = 0.034). However, *P. reflexa* was equally abundant in quadrats with a target *P. leptocoma* relative to the random expectation (Fig. 1, *t*(24) = 1.05, *P* = 0.306).

#### Niche dimensions

**Figure 2.**
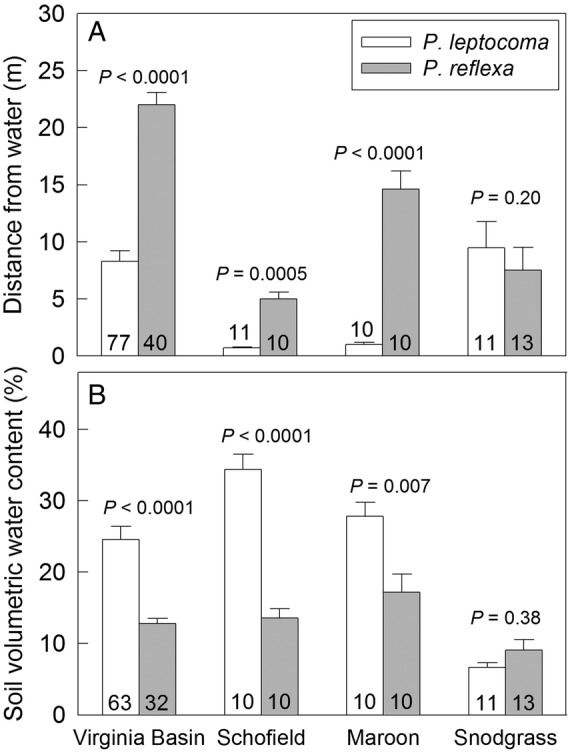
Abiotic niche characteristics of naturally occurring *P. leptocoma* and *P. reflexa* plants at each of four sites in Gunnison Co., CO, USA. (A) Mean distance from the nearest water source (± s.e.). (B) Mean volumetric water content of the soil (%) (±s.e.) measured during August 2011. Sample sizes are given on each bar, and *P*-values are shown for contrasts between pairs of means within each site.

**Figure 3.**
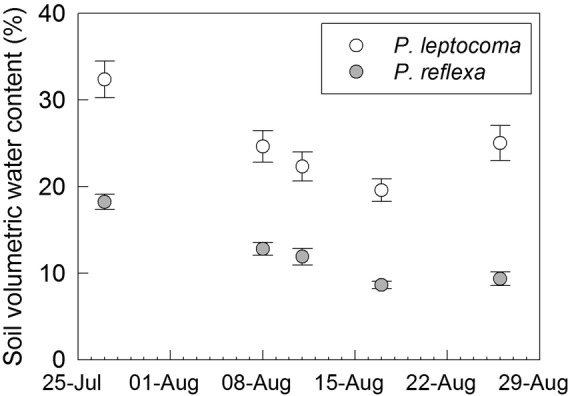
Seasonal variation in mean volumetric water content (%) (± s.e.) of the soil at naturally occurring *P. leptocoma* and *P. reflexa* plants at the Virginia Basin site in Gunnison Co., CO, USA in 2011.

*Poa leptocoma* plants typically occupied wetter microenvironments than *P. reflexa*. For instance, at the Virginia Basin site, *P. leptocoma* grew in environments an average of 8.3 m from streams, while *P. reflexa* grew in environments an average of 22.0 m from streams (Fig. 2A). Correspondingly, *P. leptocoma* sites were 92 % wetter than *P. reflexa* sites (Fig. 2B). The trend for higher soil moisture at *P. leptocoma* microsites was consistent throughout the 2011 growing season at Virginia Basin (Fig. 3).

Similar differences were observed at two of the three additional locations we surveyed (Fig. 2). At Schofield and Maroon, *P. leptocoma* grew in environments 600–1300 % closer to streams with 60–150 % higher soil moisture levels relative to *P. reflexa* environments. However, at Snodgrass Mountain, there were no significant differences between the two plant species in mean distance from a water source or mean VWC (Fig. 2). Soil moisture was lower overall at Snodgrass Mountain than at the other sites.

Colonization of roots by AM fungi was ∼15 % greater for *P. leptocoma* than for *P. reflexa*: on average, 54 % of *P. leptocoma* root intersections (±2 % s.e.) contained AM hyphae, compared with 47 % of *P. reflexa* root intersections (±2 % s.e.) (species *F*_1,57_ = 6.36, *P* = 0.015). Colonization by other types of root fungi did not differ significantly between the two *Poa* species or among sites (model *F*_5,57_ = 1.27, *P* = 0.288).

### Does endophyte presence affect the relative fitness of *P. leptocoma* versus *P. reflexa* in their putative niches?

#### Germination

**Figure 4.**
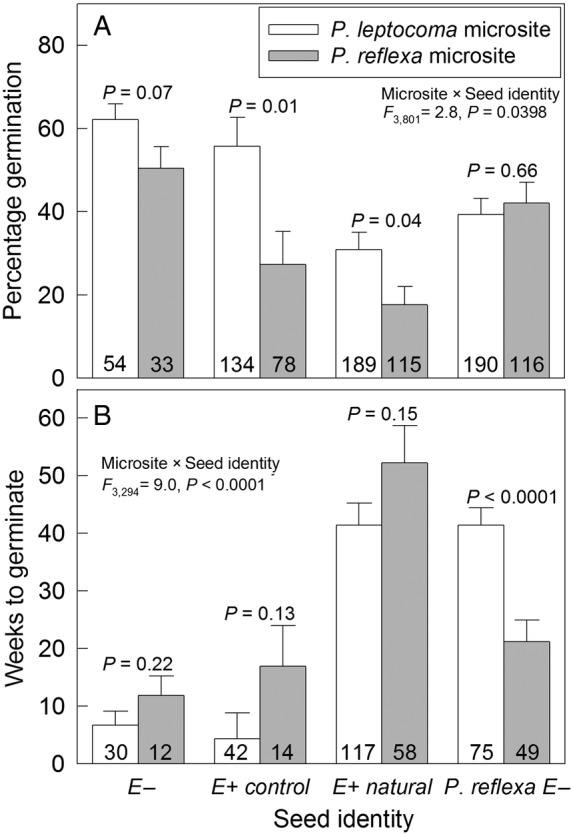
Germination responses of *P. leptocoma* and *P. reflexa* seeds in *P. leptocoma* and *P. reflexa* microsites. (A) Mean (±s.e.) percentage of seeds that germinated. (B) Mean (±s.e.) number of weeks from date of planting to date of germination. E+ natural signifies endophyte-symbiotic *P. leptocoma* seeds collected from field plants, E+ control signifies endophyte-symbiotic *P. leptocoma* seeds collected from greenhouse-grown plants, E− signifies greenhouse-grown, endophyte-free *P. leptocoma* seeds resulting from experimental disinfection, and *P. reflexa* E− seeds were collected from field plants and were always endophyte-free. Sample sizes are given on each bar, and *P*-values are shown for contrasts within each seed identity type.

Generally, each species germinated best in its own microsite, and endophyte absence/removal decreased the magnitude of this effect. In accordance with our predictions, a significantly greater percentage of endophyte-symbiotic *P. leptocoma* seeds germinated in *P. leptocoma* habitats relative to *P. reflexa* habitats (Fig. 4A, Table 1). This was the case for both greenhouse-grown (control) and field-collected (natural) E+ *P. leptocoma* seeds, which showed 100 % and 75 % higher germination rates in the *P. leptocoma* niche relative to the *P. reflexa* niche, respectively. The endophyte-free *P. leptocoma* seeds showed a much weaker difference in germination between microsites. *Poa leptocoma* endophyte-free (E−) seeds had 85–187 % higher germination in *P. reflexa* microsites than did *P. leptocoma* E+ control seeds (*P* = 0.13) or E+ natural seeds (*P* = 0.0005), respectively. For the *P. reflexa* seeds, there was no significant difference in percentage germination between *P. leptocoma* and *P. reflexa* niches, contrary to our original prediction (Fig. 4A).

**Table 1.** Results of statistical models testing the effects of seed identity (*P. leptocoma* E+ control, *P. leptocoma* E+ natural, *P. leptocoma* E− or *P. reflexa* E−), microsite type (*P. leptocoma* or *P. reflexa*) and their interaction on (a) whether or not a seed germinated, (b) the number of weeks to germination for seeds that germinated, (c) whether or not a germinated seedling survived, (d) number of weeks surviving for seedlings that died and (e) combined growth responses (number of live leaves at the time of harvest, aboveground biomass and belowground biomass) using MANOVA.

	Numerator *df*	Denominator *df*	*F*-ratio	*P*
**(a) Effect on germination (0/1)**				
Seed identity	3	801	15.5	<0.0001
Microsite	1	100	8.4	0.0045
Microsite × seed identity	3	801	2.8	0.0398
**(b) Effect on weeks to germinate**				
Seed identity	3	294	38.4	<0.0001
Microsite	1	95	0.3	0.5643
Microsite × seed identity	3	294	9.0	<0.0001
**(c) Effect on seedling survival (0/1)**				
Seed identity	3	296	6.5	0.0003
Microsite	1	95	1.9	0.1741
Microsite × seed identity	3	296	1.7	0.1700
**(d) Effect on weeks surviving for seedlings that died**				
Seed identity	3	234	5.7	0.0009
Microsite	1	92	3.7	0.0575
Microsite × seed identity	3	234	2.5	0.0599
**(e) Effect on plant growth (MANOVA)**				
Seed identity	6	118	2.40	0.0315
Microsite	3	58	0.73	0.5379
Microsite × seed identity	6	118	0.35	0.9113

In contrast, amongst seeds that germinated, *P. reflexa* seeds germinated 20 weeks earlier, on average, if planted into conspecific microsites relative to *P. leptocoma* microsites (Fig. 4B, Table 1). *Poa leptocoma* seeds with a similar maternal history (E+ natural) took an additional 31 weeks, on average, to germinate compared with *P. reflexa* seeds (Fig. 4B, *P* < 0.0001). The length of time to germinate was not affected by microsite for any of the *P. leptocoma* seed types (Fig. 4B, Table 1). In general, greenhouse-grown seeds germinated in fewer weeks than did seeds harvested from field plants (Fig. 4B).

#### Survival

**Figure 5.**
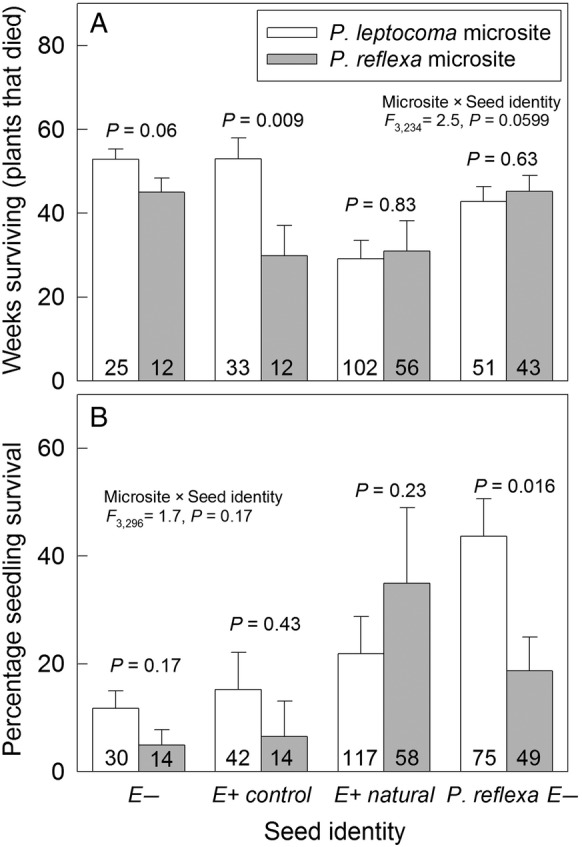
Survival responses of *P. leptocoma* and *P. reflexa* seeds in *P. leptocoma* and *P. reflexa* microsites. (A) Mean (±s.e.) number of weeks that seedlings survived (for seedlings that died). (B) Mean (±s.e.) percentage of seedlings that survived. E+ natural signifies endophyte-symbiotic *P. leptocoma* seeds collected from field plants, E+ control signifies endophyte-symbiotic *P. leptocoma* seeds collected from greenhouse-grown plants, E− signifies greenhouse-grown, endophyte-free *P. leptocoma* seeds resulting from experimental disinfection, and *P. reflexa* E− seeds were collected from field plants and were always endophyte-free. Sample sizes are given on each bar, and *P*-values are shown for contrasts within each seed identity type.

Generally, *P. leptocoma* microsites were better for seedling survival than *P. reflexa* microsites. In *P. leptocoma* microsites, *P. reflexa* seedlings showed a higher percentage survival, and *P. leptocoma* seedlings survived for a longer amount of time. Endophyte presence altered the plant survival response to microsite: in *P. reflexa* microsites, endophyte-free *P. leptocoma* seedlings survived 50 % longer than control E+ seeds (Fig. 5A, *P* = 0.058, with low power due to low germination rates in these microsites, *n* = 12). For *P. reflexa*, the effects of microsite on survival were opposite to the effects of microsite on time to germination, with 133 % higher survival of *P. reflexa* seedlings in the *P. leptocoma* microsite relative to the *P. reflexa* microsite (Fig. 5B, Table 1). Greenhouse-grown *P. leptocoma* seedlings (E− and E+ control) generally survived longer in *P. leptocoma* microsites than in *P. reflexa* microsites (Fig. 5A), while no difference was observed in length of survival between microsites for E+ natural *P. leptocoma* plants.

#### Growth

**Figure 6.**
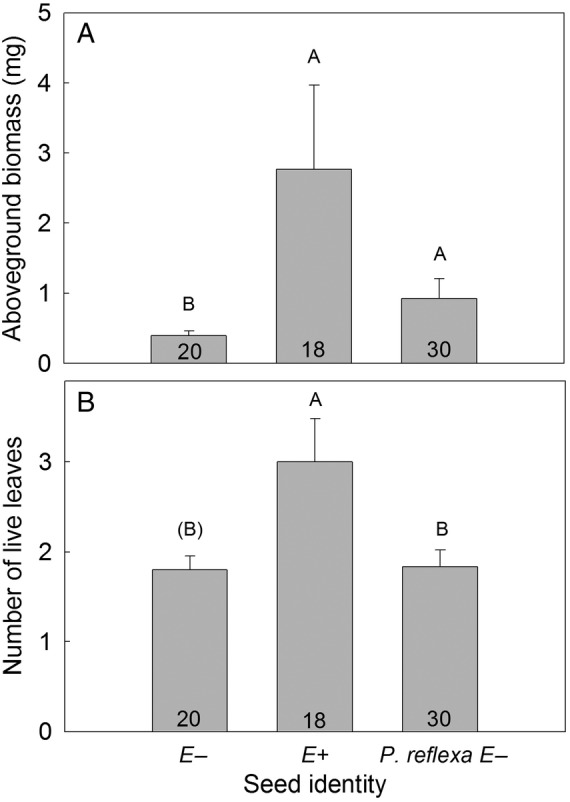
Growth responses of endophyte-free (E−) *P. leptocoma* seeds, endophyte-symbiotic (E+) *P. leptocoma* seeds and endophyte-free *P. reflexa* (*P. reflexa* E−) seeds. (A) Mean (±s.e.) aboveground biomass of plants harvested in September 2013. (B) Mean (±s.e.) number of live leaves on plants harvested in September 2013. For each growth response, means annotated with different letters significantly differed. Parentheses around a letter indicate a marginally significant difference between that mean and a mean annotated with a different letter.

Plant growth was affected only by seed identity and not by microsite (Table 1, microsite × seed identity, *P* > 0.9). Mean aboveground biomass was significantly greater for *P. leptocoma* E+ individuals relative to *P. leptocoma* E− individuals, with E+ plants having ∼600 % more biomass than E− plants (Fig. 6A). Mean aboveground biomass did not differ significantly between *P. leptocoma* E+ and *P. reflexa* plants (Fig. 6A). *Poa leptocoma* E+ individuals had a significantly greater number of live leaves, on average, relative to *P. reflexa* individuals, and a marginally greater number of live leaves relative to *P. leptocoma* E− individuals (Fig. 6B). There was no significant effect of seed identity on belowground biomass (*P* = 0.1483).

## Discussion

Our observational data indicate that *P. leptocoma* and *P. reflexa* typically occupy distinct ecological niches, with lower levels of co-occurrence than expected by chance. *Poa leptocoma* was more common in wet microsites and supported higher rates of root colonization by mycorrhizal fungi than *P. reflexa*. With regards to seed germination and seedling survival, endophyte presence appeared to constrain the distribution of *P. leptocoma* to wetter microsites, where its congener was largely absent. However, for those plants that survived their first 3 years, endophyte symbiosis yielded net benefits to plant growth, regardless of the microhabitat. Differential effects of endophyte symbiosis on different host life history stages may contribute to niche partitioning between the two congeneric plant species. Given the knowledge that endophyte effects can vary among host life history stages (Rudgers *et al.* 2012), it is premature to designate the endophyte–*P. leptocoma* interaction as a net mutualism. Here, however, we for the first time document beneficial effects of endophytes at the seedling to 3-year-old age range.

Water can be a key resource for plants in subalpine habitats (Kuramoto and Bliss 1970; Harte and Shaw 1995), but may also cause oxidative stress due to flooding, as could be the case for *P. leptocoma* individuals observed growing in running water. Our results are consistent with anecdotal observations that *P. leptocoma* inhabits wetter environments than *P. reflexa* (Barkworth *et al.* 2007). It is surprising that *P. leptocoma*, the endophyte host species, is associated with wetter areas, given that endophytes are known to promote drought tolerance in other grass species (Cheplick and Faeth 2009). However, in other systems, fungal endophyte symbiosis has been shown to enhance host tolerance to stress via pathways involving the interplay of reactive oxygen species and antioxidants (Hamilton *et al.* 2012). For instance, endophyte symbiosis appeared to convey tolerance to heat and salt stress in three plant species by lowering accumulation of reactive oxygen species in host tissues (Rodriguez *et al.* 2008). The fungal endophyte of *P. leptocoma* may confer greater tolerance of oxidative stress to its host via a similar mechanism, if indeed flooding is stressful to plants in this system.

Observations of edaphic factors, such as soil nutrient levels and pH, could yield further insight into variables that affect the distributions of *P. leptocoma* and *P. reflexa*. For instance, interspecific differences in chemical form, soil depth and timing of nitrogen uptake have been documented within plant communities, and such resource partitioning can in turn influence species coexistence and community composition (McKane *et al.* 2002; Miller and Bowman 2002; Pornon *et al.* 2007), including amongst grass species (Weigelt *et al.* 2005). Endophyte symbiosis has been found to affect the accumulation of inorganic and organic nitrogen in host grass tissues (Lyons *et al.* 1990); uptake of different nitrogen forms could be an endophyte-mediated axis of niche differentiation between the two bluegrasses examined here. Further study could also consider morphological characteristics, such as rooting depth, that may contribute to niche partitioning between the two species by allowing differential access to nitrogen and other soil resources (McKane *et al.* 2002).

Susceptibility to herbivory may be an additional biotic niche dimension that differentiates the two *Poa* species. All examined *Epichloë* populations symbiotic with *P. leptocoma* contained the *perA* gene required for the production of peramine, a documented insect deterrent (Tanaka *et al.* 2005; Schardl *et al.* 2013*b*). If the encoded *perA* gene is functional, then endophyte-symbiotic *P. leptocoma* individuals may be less susceptible to herbivory by insects relative to *P. reflexa.* Similarly, in some *P. leptocoma* populations, we found *Epichloë* species with genes for the production of the ergot alkaloid intermediate chanoclavine. While the toxicity of chanoclavine has not been well studied, many ergot alkaloids are known to be active against mammals (Schardl *et al.* 2013*a*). *Poa leptocoma* populations symbiotic with endophytes that possess genes for chanoclavine production may therefore be less affected by mammalian herbivory relative to *P. reflexa*, if the encoded genes are functional and expressed. In particular, pocket gophers (*Thomomys talpoides*), which consume both roots and shoots of grasses (Ward 1960), have been observed in the study area and may be a significant source of mortality in the system. Additional work could examine expression of the identified defensive genes, consider rates of herbivory on the two *Poa* species and manipulate herbivory levels to investigate this potential axis of niche differentiation.

While our data provide evidence of niche differences between *P. leptocoma* and *P. reflexa*, our results also indicate a certain degree of niche overlap between the two species. At one of four sites (Snodgrass Mountain), significant differences were not observed between *P. leptocoma* and *P. reflexa* in mean distance from the closest water source or in soil water content. At Snodgrass Mountain, soil moisture was overall much lower than at the other sites, and differences between *P. leptocoma* and *P. reflexa* habitats were smaller, perhaps accounting for the results obtained. Overall densities of both species were also low at this site relative to the other surveyed locations, possibly indicative of marginal habitat for both species. Other, less closely related *Poa* species are common in the general region that we sampled, and future studies might incorporate their distributions in a community phylogenetics context (Cavender-Bares *et al.* 2009).

Our results contribute to growing evidence that fungal endophyte presence can have opposing effects on different host life history stages. In accordance with our expectations, endophyte presence appeared to shift the niche of *P. leptocoma* relative to that of *P. reflexa*, affecting plant performance in microsites typical of each species. However, our results indicate that more complex dynamics are at play than we initially predicted. Endophyte symbiosis appeared to negatively affect host germination and early survival, constraining the niche of *P. leptocoma*; however, endophyte presence positively influenced the growth of plants that successfully germinated and survived, regardless of their location. Similar stage-dependent effects were observed in an experiment that removed a fungal endophyte from its host grass and showed that endophyte presence reduced host survival but increased reproduction; model predictions indicated that the beneficial effects of the endophyte on reproduction overwhelmed its negative impacts on survival (Rudgers *et al.* 2012). Our findings are also consistent with a growing number of studies that document the context-dependency of symbioses (Johnson *et al.* 1997). For instance, abiotic and biotic factors have been shown to influence the relative costs and benefits to a host plant of associations with fungal endophytes (Davitt *et al.* 2011) and AM fungi (Jones and Smith 2004; Hoeksema *et al.* 2010). Our results suggest that the outcome of a symbiosis can depend on host life history stage as well.

It is worthwhile to note that our study examined only the early life history stages of *P. leptocoma* and *P. reflexa* when testing the effects of fungal endophyte presence or absence. It is possible that the consideration of adult grasses and sexual reproduction would yield still different results regarding how the fungal endophyte contributes to niche partitioning between the two *Poa* species. An ongoing experiment is examining adult grasses and their growth and survival in *P. leptocoma* and *P. reflexa* niches. Furthermore, a competition experiment could allow for more targeted examination of how endophyte symbiosis may affect species coexistence in this system.

Further work might also consider the broader geographic distributions of the two *Poa* species. While species' range limits are expected to reflect their niche limits across continuous environments, evidence suggests that range and niche limits may or may not coincide, depending on the relative importance of factors such as dispersal, biotic interactions and temporal fluctuations (Hargreaves *et al.* 2014). In North America, the ranges of *P. leptocoma* and *P. reflexa* overlap, but *P. reflexa* has not been reported in California or Canada, whereas *P. leptocoma* has a broader distribution to both the north and west (USDA, NRCS 2014). Investigations into the respective geographic ranges of the two *Poa* species could identify whether local microsite preferences are consistent with factors that constrain range-wide species distribution limits. Transplants could be conducted outside of the range limits of each species (Hargreaves *et al.* 2014), with endophyte status manipulated in *P. leptocoma*. Recent work has shown that endophytes in the genus *Epichloë* can affect species distributions at a range-wide scale (Afkhami *et al.* 2014).

In addition, considering symbioses using phylogenetic approaches can yield insight into symbiont effects on host niche within the broader context of host evolutionary history (Poisot *et al.* 2011). For instance, examining the symbiosis between gall-inducing insects and fungi in a phylogenetic context, Joy (2013) found evidence supporting the hypothesis that symbiosis can lead to host niche expansion and diversification. Similarly, using phylogenetic approaches on an obligate pollination mutualism, Kawakita *et al.* (2010) found data suggesting that participation in a mutualism favours higher levels of specialization. However, Elias *et al.* (2008) found the opposite pattern, suggesting that mutualisms increase niche convergence. Similarly examining symbiont effects across the *Poa* clade could be informative of possible phylogenetic constraints on grass species' microsite preferences, and could shed light on whether trends documented in this study are present in other endophyte-symbiotic *Poa* species.

## Conclusions

Our study provides evidence that *P. leptocoma* and *P. reflexa* occupy different ecological niches in the habitats examined. The presence of an endophyte in *P. leptocoma* appears to play a role in partitioning the niches of the two species, perhaps by initially restricting its host's distribution to wetter microsites before positively affecting its growth. While mutualisms are known to cause shifts in partner niche dimensions, our results suggest the value of considering such effects at different partner life stages. In a broader context, our study identifies a symbiotic relationship as a potential mechanism facilitating the coexistence of two species, suggesting that symbiont effects on host niche may have community-level consequences.

## Sources of Funding

Our work was funded by the National Science Foundation (USA) (grants DEB-1354972 and DEB-1145588), the Rocky Mountain Biological Laboratory (National Science Foundation grants DBI-0753774 and OIA-0963529) and the University of New Mexico Department of Biology.

## Contributions by the Authors

M.R.K. contributed to data collection and analysis and wrote the manuscript. C.L.D. and J.A.R. designed the experiment, carried out data collection and contributed to writing the manuscript. L.R. and Y.A.C. contributed to experimental design and collected data. W.Q.H. collected data and, with N.D.C. and C.A.Y., completed DNA sequencing and phylogenetic analysis. T.H.P. completed root fungal colonization data collection and analysis. All authors edited the manuscript.

## Conflicts of Interest Statement

None declared.

## Supporting Information

The following additional information is available in the online version of this article –

**Table S1.** Locality information of sites at which *P. leptocoma* and *P. reflexa* plants were collected and/or marked, along with date of sampling, sample size and percentage of plants that were symbiotic with the fungal endophyte *E. typhina* subsp. *poae*.

**Image S1.** Photograph showing placement of plastic toothpicks around a focal, naturally occurring *Poa* individual.

**Figure S1.** Gene phylogenies placing the fungal endophyte of *P. leptocoma* within the *E. typhina* subsp. *poae* subclade.

Additional Information
