# Supplementary material for: A mutualistic endophyte alters the niche dimensions of its host plant
Source: AoB Plants. 2015 Mar 10;7:plv005. doi: 10.1093/aobpla/plv005 (PMC4354242; doi:10.1093/aobpla/plv005)
Supplement: Additional Information [file supp_7_plv005_index.html]

A mutualistic endophyte alters the niche dimensions of its host plant — Additional Information 

# A mutualistic endophyte alters the niche dimensions of its host plant

## Additional Information

Additional Information

**Files in this Data Supplement:**

- Supporting Information File 1 - Docx file
- Supporting Information File 2 - Doc file
- Supporting Information File 3 - Doc file
