# Supplementary material for: A mutualistic endophyte alters the niche dimensions of its host plant
Source: AoB Plants. 2015 Mar 10;7:plv005. doi: 10.1093/aobpla/plv005 (PMC4354242; doi:10.1093/aobpla/plv005)
Supplement: Additional Information [file supp_plv005_aobplants-14101-s02.docx]

| **Supporting Information File 1.** Table. Locality information of sites at which *Poa leptocoma* and *Poa reflexa* plants were collected and/or marked, along with date of sampling, sample size, and percentage of plants that were symbiotic with the fungal endophyte *Epichloë typhina* subsp. *poae.*  *n* indicates the number of individual plants sampled per site. Plants at the Slate River and Washington Gulch sites were used only for endophyte species characterization. | | | | | | | |
| --- | --- | --- | --- | --- | --- | --- | --- |
| **Species** | **Site** | **Latitude** | **Longitude** | **Elevation (m)** | **% Symbiotic** | ***n*** | **Date** |
| *P. leptocoma* | Cottonwood Pass | 38°48.764 | 106°24.136 | 3541 | 67 | 6 | 8/17/08 |
|  | Snodgrass Mountain | 38°55.083 | 106°59.175 | 3357 | 100 | 11 | 8/10/11 |
|  | Copper Creek 1 | 38°57.586 | 106°58.637 | 2986 | 100 | 13 | 8/15/08 |
|  | Copper Creek 2 | 38°58.112 | 106°58.048 | 3066 | 100 | 3 | 8/16/08 |
|  | Virginia Basin* | 38°58.314 | 106°58.942 | 3229 | 98 | 110 | 8/15 - 9/2/08 |
|  | Schofield Pass 1 | 39°01.329 | 107°02.938 | 3218 | 100 | 10 | 8/9/11 |
|  | Schofield Pass 2* | 39°01.461 | 107°02.960 | 3193 | 80 | 10 | 8/9/11 |
|  | Schofield Pass 3* | 39°01.497 | 107°02.850 | 3200 | 90 | 10 | 8/10/08 |
|  | Schofield Pass 4 | 39°01.489 | 107°02.851 | 3189 | 71 | 28 | 8/17/08 |
|  | Niwot Ridge | 40°02.540 | 105°34.297 | 3275 | 100 | 10 | 8/28/08 |
|  | St. Vrain 1 | 40°10.160 | 105°32.785 | 2893 | 100 | 21 | 8/26/08 |
|  | Black Lake | 40°16.103 | 105°38.373 | 3324 | 100 | 8 | 8/20/08 |
|  | St. Vrain 2* | 40°16.279 | 105°33.222 | 2907 | 90 | 10 | 8/26/08 |
|  | Slate River* | 38°93.232 | 107°05.078 | 2786 | -- | -- | 9/3/13 |
|  | Washington Gulch* | 38°93.477 | 107°00.961 | 2948 | -- | -- | 9/1/13 |
|  |  |  |  |  |  |  |  |
| *P. reflexa* | Cottonwood Pass | 38°48.764 | 106°24.136 | 3541 | 0 | 16 | 8/17/08 |
|  | Snodgrass Mountain | 38°55.083 | 106°59.175 | 3357 | 0 | 13 | 8/10/11 |
|  | Virginia Basin 3 | 38°58.187 | 106°59.001 | 3205 | 0 | 6 | 8/9/08 |
|  | Schofield Pass | 38°58.314 | 106°58.942 | 3229 | 0 | 10 | 9/1/08 |
|  | Virginia Basin 1 | 38°58.314 | 106°58.942 | 3229 | 0 | 10 | 9/2/08 |
|  | Virginia Basin 2 | 38°58.913 | 106°58.249 | 3630 | 0 | 16 | 8/11/08 |
|  | Mt. Belleview | 39°01.023 | 107°01.742 | 3630 | 0 | 9 | 8/12/08 |
|  | Schofield Pass 1 | 39°01.329 | 107°02.938 | 3218 | 0 | 10 | 8/9/11 |
|  | Schofield Pass 2 | 39°01.461 | 107°02.960 | 3193 | 0 | 10 | 8/9/11 |
|  | Niwot | 40°02.876 | 105°34.307 | 3373 | 0 | 9 | 8/28/08 |
|  | Mitchell Lake | 40°04.994 | 105°34.915 | 3214 | 0 | 10 | 8/28/08 |
|  | Black Lake | 40°16.103 | 105°38.373 | 3324 | 0 | 3 | 8/20/08 |
|  |  |  |  |  |  |  |  |
| *Indicates sites at which endophytes were characterized by DNA sequencing | | | | | | | |
