# Supplementary material for: A mutualistic endophyte alters the niche dimensions of its host plant
Source: AoB Plants. 2015 Mar 10;7:plv005. doi: 10.1093/aobpla/plv005 (PMC4354242; doi:10.1093/aobpla/plv005)
Supplement: Additional Information [file supp_plv005_aobplants-14101-s03.doc]

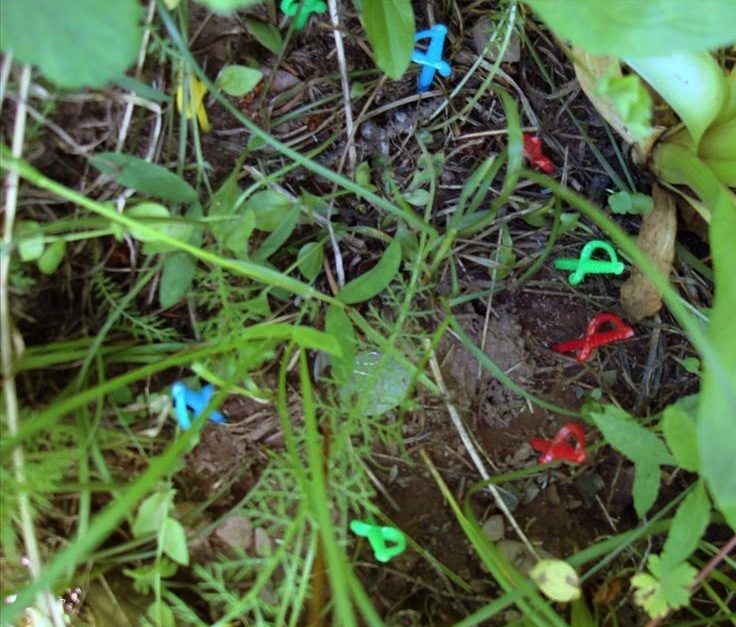


**Supporting Information File 2.** Image. Photograph showing placement of plastic toothpicks around a focal, naturally occurring *Poa* individual.
