# Supplementary material for: A mutualistic endophyte alters the niche dimensions of its host plant
Source: AoB Plants. 2015 Mar 10;7:plv005. doi: 10.1093/aobpla/plv005 (PMC4354242; doi:10.1093/aobpla/plv005)
Supplement: Additional Information [file supp_plv005_aobplants-14101-s04.doc]

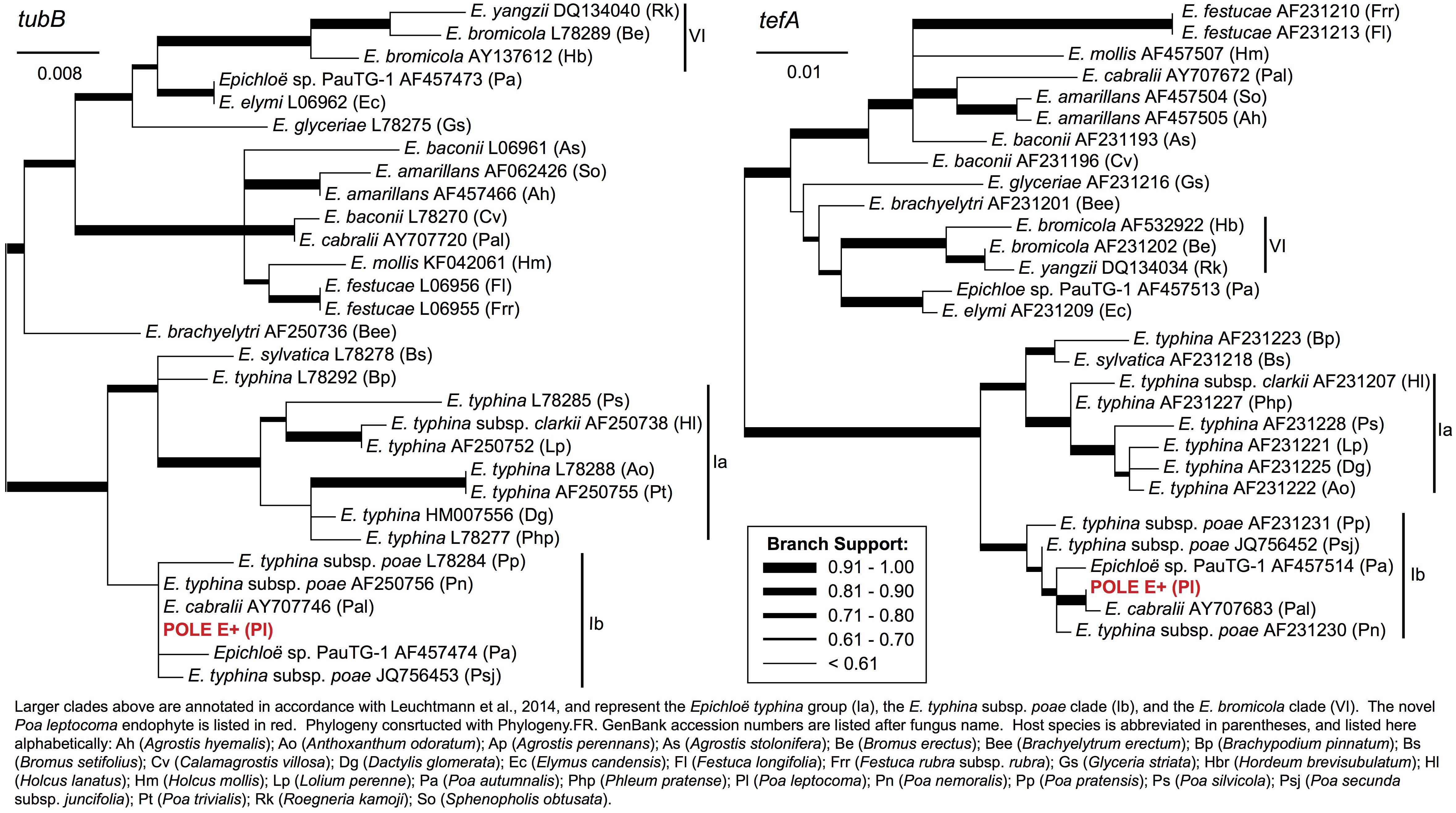


**Supporting Information File 3.** Figure. Gene phylogenies placing the fungal endophyte of *P. leptocoma* within the *Epichloë typhina* subspecies *poae* subclade. Larger clades are annotated in accordance with Leuchtmann *et al.* (2014), and represent the *Epichloë typhina* group (Ia), the *E. typhina* subsp. *poae* clade (Ib), and the *E. bromicola* clade (VI). The *P. leptocoma* (POLE E+) endophyte is listed in red. The phylogenies were constructed with phylogeny.fr. GenBank accession numbers are listed after fungus name. Host species is abbreviated in parentheses, and listed here alphabetically: Ah (*Agrostis hyemalis*); Ao (*Anthoxanthum odoratum*); Ap (*Agrostis perennans*); As (*Agrostis stolonifera*); Be (*Bromus erectus*); Bee (*Brachyelytrum erectum*); Bp (*Brachypodium pinnatum*); Bs (*Bromus setifolius*); Cv (*Calamagrostis villosa*); Dg (*Dactylis glomerata*); Ec (*Elymus candensis*); Fl (*Festuca longifolia*); Frr (*Festuca rubra* subsp. *rubra*); Gs (*Glyceria striata*); Hbr (*Hordeum brevisubulatum*); Hl (*Holcus lanatus*); Hm (*Holcus mollis*); Lp (*Lolium perenne*); Pa (*Poa autumnalis*); Php (*Phleum pratense*); Pl (*Poa leptocoma*); Pn (*Poa nemoralis*); Pp (*Poa pratensis*); Ps (*Poa silvicola*); Psj (*Poa secunda* subsp. *juncifolia*); Pt (*Poa trivialis*); Rk (*Roegneria kamoji*); So (*Sphenopholis obtusata*).
